# Profiling the microRNA signature of the peripheral sensory ganglia in experimental autoimmune encephalomyelitis (EAE)

**DOI:** 10.1186/s12974-019-1600-7

**Published:** 2019-11-15

**Authors:** Timothy N. Friedman, Muhammad Saad Yousuf, Ana Catuneanu, Mansi Desai, Camille A. Juźwik, Alyson E. Fournier, Bradley J. Kerr

**Affiliations:** 1 Neuroscience and Mental Health Institute, University of Alberta, Edmonton, Alberta T6G 2E1 Canada; 2 Department of Pharmacology, University of Alberta, Edmonton, Alberta T6E 2H7 Canada; 3 Montreal Neurological Institute, McGill University, Montreal, Quebec H3A 2B4 Canada; 4 Department of Psychiatry (NRU), University of Alberta, Edmonton, Alberta T6G 2B7 Canada; 5 Department of Anesthesiology and Pain Medicine, University of Alberta, Clinical Sciences Building, 2-150, Edmonton, Alberta T6G 2G3 Canada

**Keywords:** Micro-RNA, Pain, Inflammation, Multiple sclerosis, DRG

## Abstract

**Background:**

Multiple sclerosis is an autoimmune disease with a distinct female bias, as well as a high prevalence of neuropathic pain in both sexes. The dorsal root ganglia (DRG) contain the primary sensory neurons that give rise to pain, and damage to these neurons may lead to neuropathic pain. Here, we investigate the sex differences of the DRG transcriptome in a mouse model of MS.

**Methods:**

Next-generation sequencing was used to establish RNA and microRNA profiles from the DRG of mice with MOG_35–55_-induced EAE, a model of CNS inflammation that mimics aspects of MS. Differential expression and multiple meta-analytic approaches were used to compare expression profiles in immunized female and male mice. Differential expression of relevant genes and microRNAs were confirmed by qPCR.

**Results:**

Three thousand five hundred twenty genes and 29 microRNAs were differentially expressed in the DRG of female mice with MOG_35–55_-EAE, while only 189 genes and 3 microRNAs were differentially expressed in males with MOG_35–55_-EAE. Genes related to the immune system were uniquely regulated in immunized female mice. Direct comparison of sex within disease indicates significant differences in interferon and phagosomal pathways between the sexes. miR-21a-5p is the primary dysregulated microRNA in both sexes, with females having additional dysregulated microRNAs, including miR-122-5p.

**Conclusions:**

This study provides evidence that females are uniquely affected by MOG_35–55_-EAE and that this difference may result from additional signaling not present in the male. The altered transcriptome of females correlates with other studies finding hyperactivity of pain-sensing neurons and suggests underlying sex-specific pathways for neuropathic pain.

## Introduction

Multiple sclerosis (MS) is an autoimmune disease of the central nervous system, characterized by demyelination, disturbances in neuronal function, and progressive neurodegeneration [1]. Experimental autoimmune encephalomyelitis (EAE) is a mouse model used to study the pathophysiology of MS, where an autoimmune response is artificially induced against the myelin of the central nervous system (CNS). The model produces immune-related demyelination of the CNS [2–4]. People with MS experience altered motor function but recent estimates report that 63% of people living with MS are additionally negatively impacted by disturbances to sensation such as chronic, neuropathic pain [5]. Neuropathic pain arises from damage to the neurons involved in transmitting the “pain” signal to the brain. This damage can be localized to any part comprising the pain system, from the pain-sensing periphery to the pain-perceiving central nervous system [6].

The dorsal root ganglia (DRG) residing in the peripheral nervous system (PNS) contain primary sensory neurons including pain-sensing neurons. The precise contribution of DRG neurons and the mechanisms mediating neuropathic pain in MS and EAE remain elusive. While transcriptome dysregulation has been studied in the CNS and immune cell populations in the context of EAE [7–9], less research has been done on the PNS, a potential source of neuropathic pain in the disease. Recently, our laboratory has described differences in the development of pain states between female and male mice in the EAE model. These differences include differences in immune and neurodegenerative histochemical and oxidative stress profiles [2, 4]. These sex differences extend beyond the CNS, affecting the DRG [3]. Knowing that sex differences exist in EAE and that there are functional alterations correlated to pain, we sought to describe the transcriptional profile of the DRG in EAE from female and male mice with the disease.

MicroRNAs (miRNAs) are ~ 22 nucleotide long, single-stranded RNA molecules that repress translation of RNA species with complementary sequences [10]. miRNAs have risen to the forefront of research into the regulation of the transcriptome in pathophysiological states. It is estimated that the majority of all transcriptionally related pathways are under miRNA regulation [11]. Increasing amounts of evidence suggest that “miRNA-signatures” in various tissues can be correlated with specific disease states. As miRNAs can function as one-to-many vectors by regulating the expression of multiple genes, we were also interested to see if a “miRNA-signature” existed at the level of the DRG in addition to a functional analysis of the “transcriptome signature.” This information could yield insights into the mechanisms of pain in EAE and MS more generally.

Here, we identify EAE-related miRNAs that are differentially expressed in both male and female DRGs. We compare the two sex-specific signatures at the transcriptional level for mice with EAE. We describe the analysis of RNASeq and miRSeq datasets, and functional clustering of the differentially expressed genes and miRNAs into disease-relevant signatures. Our findings highlight extensive sex differences that must be considered with animal models of disease and identify potential targets for pain-modifying therapies in the disease.

## Methods

### MOG_35–55_-EAE generation

Female and male 6–8-week-old C57BL/6 mice were acquired from Charles River Laboratories, Canada, and habituated for 2 weeks in the housing facility with baseline handling and behavioral testing. After this period, MOG_35–55_-EAE, (hereon referred to as “EAE”) was induced by 50 μg subcutaneous injections of myelin oligodendrocyte glycoprotein (sp) (MOG_35–55_; Peptide Synthesis Facility, University of Calgary) emulsified in complete Freund’s adjuvant (CFA) at a concentration of 1.5 mg/ml. An additional intraperitoneal injection of 300 ng of pertussis toxin in 0.2 ml saline was given on the day of induction as well as 48 h later. Control (termed CFA hereon after) mice received identical injection protocols except for the lack of emulsified MOG. CFA is known to produce transient alterations in immune activity [12] but is still an effective tool for EAE induction and an appropriate control. Mice were assessed daily for clinical progression of EAE following a four-point scale: grade 0, normal mouse; grade 1, flaccid tail (disease onset); grade 2, mild hindlimb weakness with quick righting reflex; grade 3, severe hindlimb weakness with slow righting reflex; grade 4, hindlimb paralysis in one hindlimb or both. Tissue was extracted on the first day a mouse presented with clinical signs (see below) that is denoted as “onset.”

### Tissue collection and total RNA extraction

Animals were euthanized by Euthanyl® (sodium pentobarbital) overdose injected intrapertioneally. After injection, animals were monitored for level of consciousness and dissections did not proceed until no response to toe pinch or corneal contact was observed. Cardiac punctures were performed to confirm euthanization and animals were perfused with 10 mL of saline. Whole DRGs were immediately extracted and suspended in Qiazol (Qiagen Biosystems) and stored at − 80 °C for later processing. Upon collection of the full tissue set, total RNA was extracted using miRNeasy® Mini kits (Qiagen Biosystems).

### Next-generation sequencing

Total RNA was supplied to ArrayStar Inc. for sequencing. Total RNA was enriched by oligoDT magnetic beads, and library preparation was completed using KAPA Stranded RNA-Seq Library Prep Kit. Transcriptome data was acquired by sequencing on an Illumina HiSeq 4000 machine according to manufacturer’s instructions. MicroRNA data was acquired by 3′-adapter ligation with T4 RNA ligase 2 (truncated), 5′-adapter ligation with T4 RNA ligase, cDNA synthesis with RT primer, PCR amplification, and extraction and purification of ~ 130–150 bp PCR amplified fragments and sequencing on an Illumina HiSeq 2000 machine according to manufacturer’s instructions.

### Analysis of differential expression

Both RNASeq and miRSeq differential expression was investigated through the R [13] package DESeq2. Briefly, raw transcript sequences from NGS were aligned to the UCSC “mm10” database of known genes using the Genomic Features package function “exonsBy.” miR read counts were directly uploaded into R. The resulting data structures were processed through the DESeq2 package following the author’s instructions found in their published vignette [14–16]. The results of each contrasting analysis (e.g., M EAE vs. M CFA) were published in a tabular format for usage in subsequent analyses. Genes were considered significantly differentially expressed using the threshold of *q* <  0.1 after Benjamini-Hochberg correction. Clustergram and PCA analyses were performed using MATLAB (MATLAB and Statistics Toolbox Release 2017b, The MathWorks, Inc., Natick, MA, USA).

### Ontological analysis with GOrilla/REViGo

Single gene lists ranked by *p*-adjusted values were analyzed with the GOrilla [17] web software, using a *p* value threshold of 10E−9. Significant GO terms from each category (process, function, component) were then processed through REViGO [18] to screen for redundant terminology.

### Functional analysis with ingenuity pathway analysis

Activation patterns of canonical pathways were analyzed through the use of IPA (QIAGEN Inc., https://www.qiagenbioinformatics.com/products/ingenuity-pathway-analysis) [19]. Briefly, gene lists ranked by *p*-adjusted values were analyzed using the “Core Analysis” feature using default parameters and a *p*-adjusted cutoff of 0.1.

### qPCR validation

For validation of DEmiRs, 50 ng of total RNA was reverse transcribed using the miScript II RT Kit (Qiagen Biosystems). miRs were detected with the Mm_miR-21_2 and Mm_miR-122a_1 miScript Primer Assays (Qiagen Biosystems) using the miScript SYBR Green PCR Kit (Qiagen Biosystems). Values detected were standardized to sno72. For validation of DEGs, 100 ng of total RNA was processed with DNase I (Invitrogen) followed by reverse transcription using Superscript III (Invitrogen). Transcripts were detected using the following primers: Ppia: RT^2^ qPCR Primer Assay for Mouse Ppia (Qiagen Biosystems), C3: Forward, GAGGCACATTGTCGGTGGTG, Reverse, CCAGGATGGACATAGTGGCG, C5ar1: Forward, ATGGACCCCATAGATAACAGCA, Reverse, GAGTAGATGATAAGGGCTGCAAC, Stat1: RT^2^ qPCR Primer Assay for Mouse Stat1 (Qiagen Biosystems) RT^2^ qPCR Primer Assay for Mouse Tlr7 (Qiagen Biosystems), RT^2^ qPCR Primer Assay for Mouse Tlr8 (Qiagen Biosystems), and RT^2^ qPCR Primer Assay for Mouse Trem2 (Qiagen Biosystems). Transcripts detected were standardized to Ppia values. All qPCR reactions were 20 uL volumes utilizing a StepOnePlus machine (Applied Biosystems).

### qPCR statistical analysis

Statistical analysis was performed using Graphpad Prism 6.01. qPCR analysis utilized two-way ANOVA testing with Sidak’s correction for multiple testing. qPCR statistics were performed on untransformed values but plotted as linearized values for ease of visualization. Alpha was set to 0.05 for all testing.

## Results

### RNASeq

**Fig. 1:**
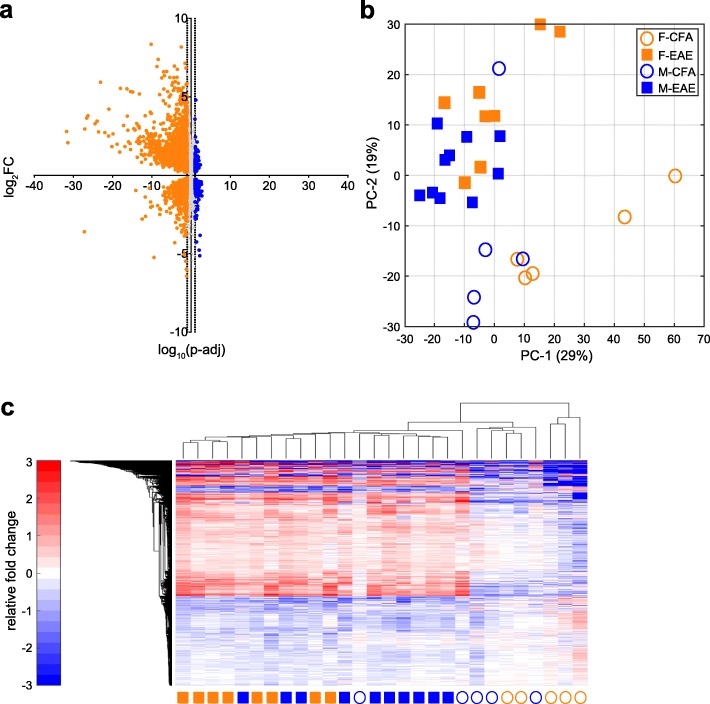
EAE primarily affects female DRGs over males at the transcriptional level. **a** Joined volcano plots of DEGs; female DEGs = 3520, male DEGs = 189. Vertical dotted lines represent log_10_(*q* = 0.1) as a cutoff threshold for significance. **b** Principal component analysis suggests separation of the EAE and CFA DRGs when analyzed by all mRNA sequencing data. Top two components are plotted. **c** Heatmaps of all DEGs in EAE further visualize the clustering of EAE. Values are Reads/Totals Reads normalized to the average of the CFA condition. DEG = differentially expressed gene

To detect a transcriptional signature within the DRG of EAE mice, we performed next-generation sequencing (NGS) of DRGs taken from mice at the onset of the disease when pain hypersensitivity is evident [2, 3, 20]. Roughly 20,000 genes were detected by sequencing. We observed gross differential expression of genes (DEGs) in mice subjected to EAE both in female (3520 DEGs) and male (189 DEGs) mice relative to their sex-specific CFA controls (Fig. 1a, Additional file 1: Table S1 and Additional file 2: Table S2) (Wald test; *q* <  0.1). To visualize variation in animal profiles and expression patterns, we performed a principal component analysis of all animals by all genes. This unbiased approach projects numerous complex variables into simpler dimensions, allowing for appreciation of relatedness between samples based on their proximity. The first two components captured almost half of the total variation within the entire sequencing set, with 29 and 19%, respectively (Fig. 1b). Cluster analysis (MATLAB clustergram) of all animals by the sum of female and male DEGs showed high specificity for EAE over CFA control, but minimal specificity for sex (Fig. 1c).

### Detection of EAE-related sex-specific signatures

**Fig. 2:**
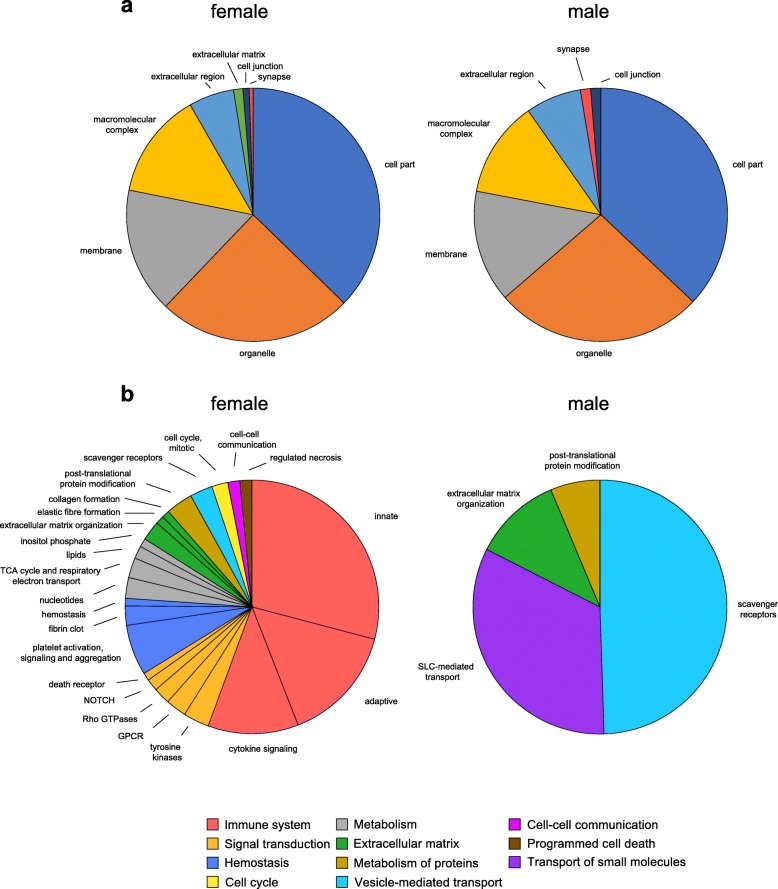
Female and male DRGs exhibit unique functional RNA signatures. **a** Both sexes show similar PANTHER Pathways GO cell compartment terms. **b** However, REACTOME pathways of female and male DEGs show a distinct profile, suggesting that the function of each set of DEGs may be different. Color legends represent grouped terms under the REACTOME hierarchy. Wedges are scaled to # DEGs in pathway/# genes defined in the respective pathway

To contextualize the significant genes into functional groups from known ontologies, we enlisted the DAVID [21], REACTOME [22], GOrilla [17], REViGO [18], and PANTHER [23–25] algorithms. Analysis of DEGs generated by DESeq analysis for significantly enriched GOslim (Gene Ontology) terms for Cell Compartment (Fig. 2a) showed a similar pattern of gene regulation for females and males. The Cell Compartment analysis references genes known to localize in specific regions of the cell. While many terms were identical in enrichment (i.e., “membrane”), further analysis of GO terms revealed some unique variations in Cell Compartment, as males included enrichment in mitochondrial terms whereas females were enriched in vesicular related terms (Tables 1 and 2). Remarkably, analysis of DEGs by REACTOME pathways to cluster molecular events revealed a striking sex-specific signature (Fig. 2b). The majority of pathways enriched in females such as immune system-related pathways were completely absent in males. Male EAE mice contained significant enrichment of a single unique term: “SLC-mediated transport,” with all others sharing overlap with females.

**Table 1:** GOrilla/REViGO analysis of differentially expressed genes in female EAE. Top 10 non-dispensable terms are reported

REViGO term	-log(*p* value)	Dispensability	Frequency (%)
Extracellular region	23.8069	0	2.38
Cell surface	19.1549	0	0.24
Membrane	27.0114	0	61.59
Vesicle	9.279	0	1.36
Extracellular region part	30.5186	0	1.31
Side of membrane	20.0101	0	0.21
Membrane region	12.4225	0.037	0.12
External side of plasma membrane	19.3224	0.046	0.06
Cell part	20.251	0.087	52.39
Cytoplasmic part	14.284	0.214	12.66

**Table 2:** GOrilla/REViGO analysis of differentially expressed genes in male EAE. Top 10 non-dispensable terms are reported

REViGO term	-log(*p* value)	Dispensability	Frequency (%)
Membrane	19.9136	0	61.59
NADH dehydrogenase complex	12.2565	0	0.04
Organelle	9.8665	0	20.79
Extracellular region part	11.5017	0	1.31
Respiratory chain	9.8239	0.035	0.30
Cell surface	11.9872	0.045	0.24
Mitochondrial membrane part	11.4377	0.053	0.38
Cell part	17.4437	0.093	52.39
Receptor complex	9.9747	0.233	0.12
Cytoplasmic part	16.0414	0.261	12.66

### Validation of RNASeq

**Fig. 3:**
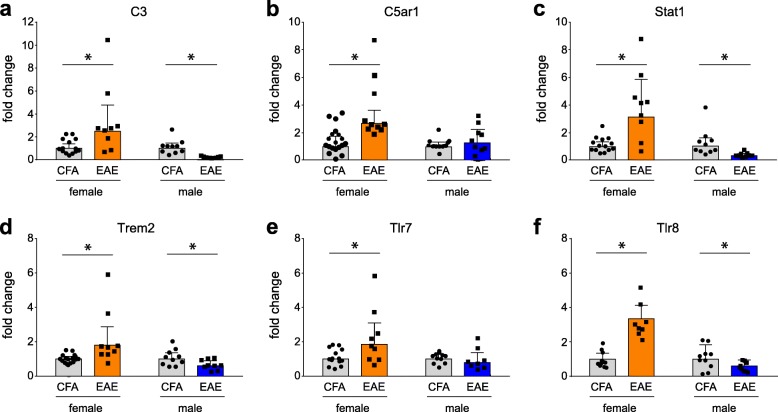
Validation of DEGs by qPCR. Bar graphs of **a** C3, **b** C5ar1, **c** Stat1, **d** Trem2, **e** Tlr7, and **f** Tlr8. *p <  0.05, two-way ANOVAs with Sidak’s multiple comparison test. Bars indicate geometric mean with 95% confidence interval

To confirm the differential expression of transcripts indicated by RNASeq analysis and of special interest through subsequent meta-analyses, we performed qPCR on relevant target genes of interest (Fig. 3). Validation targets were chosen based on criteria of differential expression between EAE and CFA condition, differential expression between female and male EAE conditions, and inclusion in significant canonical pathways of Ingenuity Pathway Analysis. (Additional file 1: Table S1, Additional file 2: Table S2, Additional file 5: Table S5 and Additional file 6: Table S6). In females, we confirmed the upregulation of *C3*, *C5ar1*, *Stat1*, *Trem2*, *Tlr7*, and *Tlr8*, in concordance with RNASeq predications. Interestingly, we also detected significant downregulation of *C3*, *Stat1*, *Trem2*, and *Tlr8* in male EAE compared to their control (Table 3).

**Table 3:** qPCR statistics for DEGs and DEmiRs

DEG/DEmiR	Female		Male		Main effect	*p* value	2° effect	*p* value
	Fold change	Adjusted *p* value	Fold change	Adjusted *p* value				
*C3*	2.48	0.0029	0.17	< 0.0001	Sex	< 0.0001	Disease	0.0384
*C5ar1*	2.69	0.0079	1.23	0.8121	Disease	0.0170	~	~
*Stat1*	3.13	< 0.0001	0.33	0.0005	Sex	< 0.0001	~	~
*Trem2*	1.80	0.0072	0.61	0.0375	Sex	0.0004	~	~
*Tlr7*	1.85	0.0182	0.80	0.6256	Sex	0.0174	~	~
*Tlr8*	2.82	< 0.0001	0.60	0.0478	Sex	< 0.0001	~	~
*miR-21*	3.78	< 0.0001	4.76	< 0.0001	Disease	< 0.0001	~	~
*miR-122*	4.36	0.0232	0.22	0.1188	Sex	0.0037	~	~

### miRSeq

**Fig. 4:**
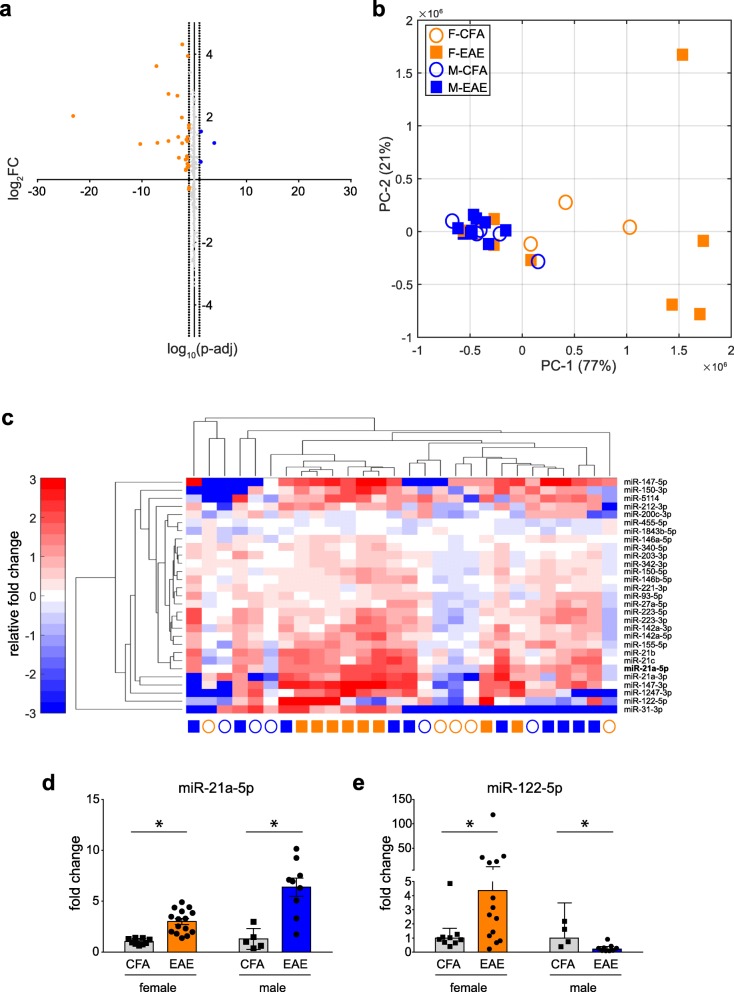
miR-21a-5p is the candidate miR for EAE in females and males. **a** Joined volcano plots of DEmiRs; female DEmiRs = 29, male DEmiRs = 3. Vertical dotted lines represent log_10_(*q* = 0.1) as a cutoff threshold for significance. **b** Principal component analysis shows minimal separation of EAE and CFA animals by all miR sequencing data. Top two components are plotted. **c** Heatmaps of all DEmiRs in EAE. Values are Reads / Totals Reads normalized to the average of the CFA condition. **d** qPCR validation of miR-21a-5p in female and male DRGs. **e** qPCR validation of miR-122-5p in female and male DRGs. *p <  0.05, two-way ANOVAs with Sidak’s multiple comparison test. Bars indicate geometric mean with 95% confidence interval. DEmiR = differentially expressed miRs

In addition to sequencing for gene coding fragments, we acquired NGS data for almost 2000 microRNAs (miRNAs). Differential expression was determined using an identical methodology to the RNASeq data above. We detected 29 differentially expressed miRNAs (DEmiRs) in the DRG of EAE females relative to CFA female controls, but only 3 DEmiRs in EAE males (Fig. 4a, Additional file 3: Table S3 and Additional file 4: Table S4) (Wald test; *q* <  0.1). All DEmiRs detected in males (miR-21a-5p, miR-21c, and miR-142a-5p) were similarly detected in females and displayed similar patterns of upregulation. Multidimensional variation was visualized by PCA analysis of all expressed miRNAs (Fig. 4b), and cluster analysis of DEmiR expression values was carried out in a similar manner to the RNAseq data set (Fig. 4c). We did not, however, identify a separation by disease by miRSeq variation unlike the RNASeq suggesting that any disease-related alterations may be more subtle.

### Differences in transcriptional signatures by sex within EAE

We next wanted to directly compare females and males with EAE to determine the effect of sex on the transcriptional profiles in the DRG. To accomplish this, we first compared the groups of “Female EAE” and “Male EAE” to find ~ 6000 DEGs. To account for genes differentially expressed as a result of sex (such as Y-linked genes), we removed DEGs from an “all Female” vs. “all Male” comparison, leaving 869 DEGs (Additional file 5: Table S5). We were interested in the functional significance of the DEGs and relied upon Ingenuity Pathway Analysis to investigate hypothetical activation of known pathways. Direct comparison yielded terminology related to metabolism (i.e., LXR/RXR activation), phagocytosis (i.e., acute phase response), and interferon signaling (Table 4 and Additional file 6: Table S6). Negative log(*p* values) correspond to increasing levels of significance (−log(≤ 0.05) ≥ 1.3), ratios represent the proportion of DEGs/# of genes in canonical pathway, and *z*-scores represent the predicted activation state of the pathway, with positive and negative being activated and inhibited, respectively. The heightened activation state of these pathways suggests an increased amount of immune-related inflammation in the DRGs of females compared to males with EAE.

**Table 4:** Canonical pathways differentially expressed between female vs. male EAE

Ingenuity canonical pathways	-log(*p* value)	Ratio	*z*-score
LXR/RXR activation	5.06	0.145	2.673
Acute phase response signaling	4.44	0.116	1.155
Neuroinflammation signaling pathway	3.57	0.0871	0.229
Complement system	3.54	0.212	1.342
FXR/RXR activation	3.12	0.113	N/A
Phagosome formation	2.88	0.107	N/A
Cytotoxic T lymphocyte-mediated apoptosis of target cells	2.39	0.185	1
Hepatic fibrosis/hepatic stellate cell activation	2.21	0.0838	N/A
GP6 signaling pathway	2.14	0.0909	0.302
Interferon signaling	2.12	0.161	2.236

### Validation of miRSeq

**Fig. 5:**
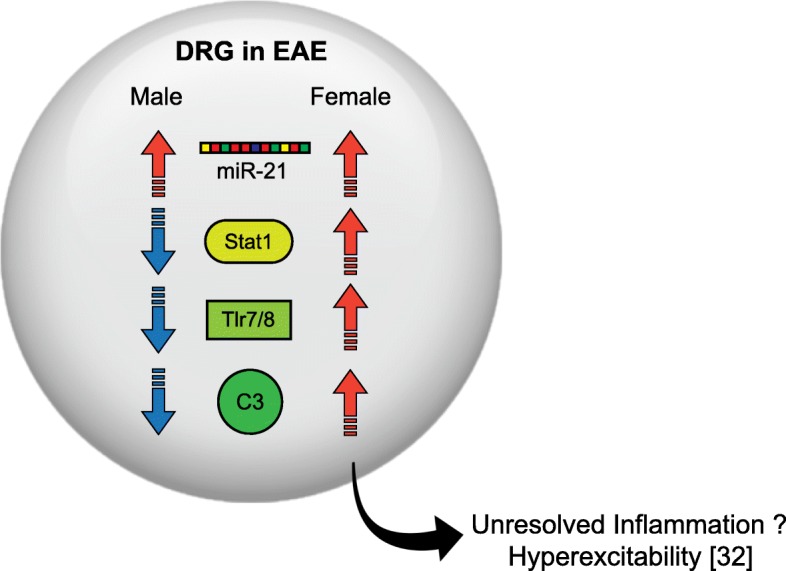
Schematic of a hypothetical DRG in EAE. Although we observe increased miR-21 in both sexes, only females display the increased inflammatory signaling (see reference)

We next wanted to validate the differential expression of miRNAs in both females and males with EAE. We identified miR-21a-5p as the most significantly dysregulated miRNA in both female and male EAE relative to respective CFA control, as well as miR-122-5p as being significantly dysregulated in female EAE alone. We validated that miR-21a-5p (hereon referred to as miR-21) was increased in both female and male EAE mice relative to CFA controls by 3.78 and 4.76-fold, respectively (Fig. 5a, b). There was no significant difference in the level of miR-21 upregulation between the sexes (2-way ANOVA, disease main effect F_1,34_ = 79.21, *p* <  0.0001, followed by Sidak’s multiple comparison test for disease within females, *t* = 6.794, *q* <  0.0001, and males, *t* = 6.021, *q* <  0.0001). miR-122-5p (hereon referred to as miR-122) was confirmed to be significantly upregulated in females only relative to CFA female controls by 4.36-fold, with a significant difference between the sexes (2-way ANOVA, sex main effect F_1,32_ = 9.803, *p* = 0.0037, followed by Sidak’s multiple comparison test for disease within females, *t* = 2.675, *q* = 0.0232, and males, *t* = 1.940, *q* = 0.1188) (Fig. 5c, d).

## Discussion

EAE is a complex disease that primarily targets the CNS. Less is known about how the PNS is affected in EAE. We have shown that a disease-related transcriptional signature is detectable in the DRG of EAE mice and that sex is an important factor in the characterization of this signature. To accomplish this, we utilized next-generation sequencing for mRNA and miRNAs from the DRG at the clinical onset of EAE and profiled the differentially expressed genes and miRNAs to acquire a functional signature for each sex. The identification of functional signatures for complex disease states or comorbidities such as pain is relevant for prompting strategic experimental design and stratifying future therapies.

### Detecting the signature

We first wanted to describe the transcriptome of our animals, considering sex as a factor. Sex differences have been previously described in immunity [26–28] and inflammatory responses [29, 30], and both of these processes play a key role in our disease model. Indeed, we found extensive differential expression of the transcriptome in female EAE mice but only small amounts of differentially expressed genes (DEGs) in male EAE mice. Large changes in gene expression have been reported in the DRG after peripheral nerve injury, and these changes have been associated with neuropathic pain behaviors [31, 32]. This supports previous evidence from our laboratory, indicating that female but not male DRG neurons are hyper-responsive at the onset of EAE [3]. In addition to these differences in the quantity of DEGs, we also found major differences in the functional signature of this altered transcriptional profile. Our data indicates that there are extensive immune and cell-signaling-related processes disturbed in the female DRGs, a potential by-product of immune cells transiently localized there [33].

### Sex-specific disease effects

Comparing the sexes directly, we noted the appearance of phagosomal activation pathways, complement, and interferon signaling as being differentially affected in female EAE versus their CFA controls (Table 3). The phagosome-related terms contained references to DEGs such as *Trem2*, *Csf1r*, *Tlr7*, and most of the complement system. Taken together, this data indicates sex-specific dysfunction of inflammatory activity in phagocytic cells. Laffont et al. have reported increased interferon responses of estrogen activated human plasma dendritic cells to TLR7 ligands, suggesting that this mechanism may be sex-hormone linked [34]. *Tlr7* is an X-linked gene and this may account for the sex-bias we observed, as X chromosome genes may escape silencing.

Interferon signaling is an approved target for MS therapy, as beta-interferons have been utilized in relapsing-remitting MS since the late 1990s [35]. Although the effects of interferon inhibitors are modest, all clinical trials conducted to date have been with mixed female and male populations. The possibility of female specific interferon dysregulation raises the possibility of higher efficacy in a subset of the clinical population. We validated the female-EAE-specific increased expression of *Stat1*—a downstream effector of types I and II interferons—suggesting that interferon signaling is activated in these animals. In male EAE mice however, *Stat1* levels were significantly reduced suggestive of a silencing of interferon signaling. As beta-interferon therapy would activate these pathways, it follows that their effect may have differential effects based on sex.

Activation of interferon signaling through *Stat1* typically results in increased inflammation and enhanced immune activity [36]. While these pathways do seem to be activated in the diseased group of females, it is apparent that it is not the case for the males. The high activation of interferon-signaling and *Stat1* in females is complemented by a similar increase in transcripts associated with inflammatory macrophages, including most members of the complement system. Complement proteins are involved with the recruitment and phagocytic capacity of innate immune cells [37, 38]. Based on our transcriptomic data, females may be poised to specifically respond to interferon stimulation, resulting in heightened phagocytic activity in the DRG in response to EAE.

### Accounting for the signature

To account for the transcriptomic changes observed in EAE, we also sequenced the miR-transcriptome. We observed a small amount of overlap between the detected DEmiRs in each sex, corresponding to miRNAs previously linked to EAE and the MS literature: miRs-21 [39, 40] and − 142 [41]. The most significant miRNA for both sexes, miR-21, was both highly expressed and confirmed to be significantly upregulated in both female and male EAE DRGs. MS brain lesions have been shown to be enriched with miR-21 in patients with MS but not neuromyelitis optica (NMO) [42]. NMO is an autoimmune disease with similar symptomatology, potentially indicating that this signature is specific to MS/EAE pathology. Therefore, miR-21 may be suitable as a marker of EAE. miR-21 has been well studied in the context of cancer, giving rise to its label of an “oncomiR” [43–45]. Interest in miR-21 as a regenerative marker is also emerging with evidence that it is pro-regenerative [46] and immunomodulatory [47]. Macrophages uptake miR-21 laden exosomes after peripheral nerve injuries [48] where it can act as a direct ligand for Toll-like receptors 7 and 8 [49, 50]. Interestingly, miR-21 is affected by sex hormones, containing a direct response element for androgens [51] and directly affected by estradiol signaling [52, 53]. As miR-21 was similarly upregulated in both sexes, we additionally screened for miRNAs unique to sex. miR-122 was only upregulated in the diseased female DRG. miR-122 is primarily described for its expression in the liver; however, it is known to increase in the circulation after injury [54, 55] and may interact with type I interferon signaling [56]. Together with the transcriptomic signatures, our data indicates that the female DRGs may be uniquely affected by the epigenetic regulation of miRs-21 and -122. Although EAE is canonically not thought of as a PNS disease, the presence of the typical “MS-miR-signature” indicates that female DRGs may be affected concordantly with CNS lesions.

## Conclusion

To our knowledge, this is the first presentation of transcriptome and miR-transcriptome dysregulation in DRGs using the EAE mouse model. We have shown that the miR-signature is primarily defined by miR-21, bearing similarity to previously described findings in the spinal cord and serum of mice with EAE, and MS lesions. We present evidence of a female-biased dysregulation of both “omic” analyses, the presence of distinct functional transcriptomic signatures for each sex, and that interferon signaling and phagosome function may be indicated for future analysis. Acknowledging fundamental differences between the sexes is not only important for experimental design, but for the development of potential sex-specific therapies. Our study provides a platform to study sex-specific changes in the DRG of EAE and the role that miRNAs may have on their transcriptome.

## Supplementary information

**Additional file 1: Table S1.** Spreadsheet of RNASeq differential expression analysis between EAE and CFA condition in females.

**Additional file 2: Table S2.** Spreadsheet of RNASeq differential expression analysis between EAE and CFA condition in males.

**Additional file 3: Table S3.** Spreadsheet of miRSeq differential expression analysis between EAE and CFA condition in females.

**Additional file 4: Table S4.** Spreadsheet of miRSeq differential expression analysis between EAE and CFA condition in males.

**Additional file 5: Table S5.** Spreadsheet of RNASeq differential expression analysis between EAE conditions of females and males.

**Additional file 6: Table S6.** Spreadsheet of Ingenuity Pathway Analysis of differentially expressed genes between female and male EAE conditions.

## Data Availability

All data generated or analyzed during this study are included in this published article and its supplementary information files.

## Abbreviations

CFA: Complete Freund’s adjuvant
CNS: Central nervous system
DEGs: Differential expression of genes
DRG: Dorsal root ganglia
EAE: Experimental autoimmune encephalomyelitis
GO: Gene Ontology
miRNAs: MicroRNAs
miRseq: MicroRNA sequencing
MOG: Myelin oligodendrocyte glyocoproteinn
MS: Multiple sclerosis
NGS: Next-generation sequencing
PNS: Peripheral nervous system
RNAseq: RNA sequencing
